# Association of Care Environment With HIV Incidence and Death Among Orphaned, Separated, and Street-Connected Children and Adolescents in Western Kenya

**DOI:** 10.1001/jamanetworkopen.2021.25365

**Published:** 2021-09-16

**Authors:** Paula Braitstein, Allison DeLong, David Ayuku, Mary Ott, Lukoye Atwoli, Omar Galárraga, Edwin Sang, Joseph Hogan

**Affiliations:** 1 Division of Epidemiology, Dalla Lana School of Public Health, University of Toronto, Toronto, Canada; 2 Department of Epidemiology and Medical Statistics, School of Public Health, College of Health Sciences, Moi University, Eldoret, Kenya; 3 Academic Model Providing Access to Healthcare (AMPATH) Program, Eldoret, Kenya; 4 Department of Biostatistics, Brown University School of Public Health, Providence, Rhode Island; 5 Department of Mental Health and Behavioral Sciences, School of Medicine, College of Health Sciences, Moi University, Eldoret, Kenya; 6 Department of Pediatrics, Faculty of Medicine, Indiana University, Indianapolis; 7 Dean’s Office, Aga Khan University Medical College, East Africa, Nairobi, Kenya; 8 Department of Health Services, Policy and Practice, Brown University School of Public Health, Providence, Rhode Island

## Abstract

**Importance:**

In 2015, there were nearly 140 million orphaned children globally, particularly in low- and middle-income regions, and millions more for whom the street is central to their everyday lives. A total of 16.6 million children were orphaned because of deaths associated with HIV/AIDS, of whom 90% live in sub-Saharan Africa. Although most orphaned and separated children and adolescents in this region are cared for by extended family, the large number of children requiring care has produced a proliferation of institutional care. Few studies have investigated the association between care environment and physical health among orphaned and separated youths in sub-Saharan Africa.

**Objective:**

To examine the association of care environment with incident HIV and death among orphaned and separated children and adolescents who were living in charitable children’s institutions, family-based settings, and street settings in western Kenya over almost 10 years.

**Design, Setting, and Participants:**

The Orphaned and Separated Children’s Assessments Related to Their Health and Well-Being (OSCAR) project was an observational prospective cohort study conducted in Uasin Gishu County, Kenya. The cohort comprised 2551 orphaned, separated, and street-connected children from communities within 8 administrative locations, which included 300 randomly selected households (family-based settings) caring for children who were orphaned from all causes, 19 charitable children’s institutions (institutional settings), and a convenience sample of 100 children who were practicing self-care on the streets (street settings). Participants were enrolled from May 31, 2010, to April 24, 2013, and were followed up until November 30, 2019.

**Exposures:**

Care environment (family-based, institutional, or street setting).

**Main Outcomes and Measures:**

Survival regression models were used to investigate the association between care environment and incident HIV, death, and time to incident HIV or death.

**Results:**

Among 2551 participants, 1230 youths were living in family-based settings, 1230 were living in institutional settings, and 91 were living in street settings. Overall, 1321 participants (51.8%) were male, with a mean (SD) age at baseline of 10.4 (4.8) years. Most participants who were living in institutional (1047 of 1230 youths [85.1%]) or street (71 of 91 youths [78.0%]) settings were double orphaned (ie, both parents had died). A total of 59 participants acquired HIV infection or died during the study period. After adjusting for sex, age, and baseline HIV status, living in a charitable children’s institution was not associated with death (adjusted hazard ratio [AHR], 0.26; 95% CI, 0.07-1.02) or incident HIV (AHR, 1.49; 95% CI, 0.46-4.83). Compared with living in a family-based setting, living in a street setting was associated with death (AHR, 5.46; 95% CI, 2.30-12.94), incident HIV (AHR, 17.31; 95% CI, 5.85-51.25), and time to incident HIV or death (AHR, 7.82; 95% CI, 3.48-17.55).

**Conclusions and Relevance:**

In this study, after adjusting for potential confounders, no association was found between care environment and HIV incidence or death among youths living in institutional vs family-based settings. However, living in a street setting vs a family-based setting was associated with both HIV incidence and death. This study’s findings suggest that strengthening of child protection systems and greater investment in evidence-based family support systems that improve child and adolescent health and prevent youth migration to the street are needed for safe and beneficial deinstitutionalization to be implemented at scale.

## Key Points

**Question:**

What is the association of care environment with incident HIV and death among orphaned, separated, and street-connected children and adolescents in western Kenya?

**Findings:**

In this cohort study of 2551 orphaned, separated, and street-connected youths in western Kenya, living in an institutional environment was not associated with either death or incident HIV compared with living in a family-based setting. However, living in a street setting was associated with a higher rate of incident HIV and death compared with living in a family-based setting.

**Meaning:**

This study’s findings suggest that alternatives for orphaned, separated, and street-connected youths in low-income regions are needed when designing public policies for the care of vulnerable children, including deinstitutionalization, to ensure that the rights and well-being of children and adolescents are upheld.

## Teaser

This cohort study examines the association of care environment with HIV incidence and death among orphaned and separated youths living in institutional, family-based, and street settings in western Kenya over an almost 10-year period.

## Introduction

The United Nations Children’s Fund (UNICEF) defines an orphan as a child younger than 18 years who has lost 1 or both parents to death associated with any cause.^1^ There were approximately 140 million orphaned children globally in 2015, including 52 million children in Africa.^1^ A total of 15.1 million children worldwide were orphaned by both parents (ie, double orphaned), and 16.6 million children were orphaned because of parental deaths associated with HIV/AIDS, of whom 90% live in sub-Saharan Africa.^1,2^ Although most orphaned and separated children and adolescents are cared for by a surviving parent or extended family members,^1^ the large number of children requiring care on the subcontinent has produced a proliferation of institutional care settings (eg, orphanages and rescue centers). Recent research has estimated that 650 000 to 1.38 million orphaned and separated children and adolescents live in institutional care environments in sub-Saharan Africa.^3^

The appropriateness of institutional care for orphaned and separated children and adolescents has been challenged because data have suggested unfavorable short- and long-term physical and mental health outcomes among children living in these environments.^4,5^ Studies have reported deficits and delays in physical growth, psychological health, and cognitive and developmental outcomes among children living in institutional settings. In response, some international organizations have advocated for a global policy of deinstitutionalization.^4,6,7^ Most studies supporting this policy have originated in eastern Europe, with relatively few from sub-Saharan Africa and other low- and middle-income countries.^4,5^ Sub-Saharan Africa has had the highest dependency ratios (ie, the number of children aged ≤15 years and adults aged ≥65 years per 100 persons of working age) worldwide for decades, and these ratios continue to increase.^8,9^ Sub-Saharan Africa also has the largest population of people living in extreme poverty, and the World Bank has estimated that 87% of the world’s lowest-income population is expected to live in that region by 2030.^10^ Numerous studies from sub-Saharan Africa have found that these constraints on families and traditional caregiving may be associated with worse educational, health, and other outcomes among orphaned children, which have been widely documented in family-based settings.^11,12,13,14,15,16,17,18,19^

Thus, despite concerns regarding the safety and care of children in institutions, there is increasing discourse about whether and how institutions might, if well designed, managed, and monitored, be able to provide a safety net or last option for children in low- and middle-income countries when other care options may be unsafe or unavailable, such as in sub-Saharan Africa, where the foster care system is not well developed or monitored.^20,21,22^ Contextually relevant, rigorous, and longitudinal data are needed to inform safe and beneficial care and to support policies and interventions for orphaned and separated children and adolescents, including safe and beneficial deinstitutionalization.

Few studies have investigated the association between care environment and physical health among orphaned and separated children and adolescents in sub-Saharan Africa.^23,24,25,26,27^ We sought to compare the incidence of HIV and death among orphaned and separated children and adolescents living in family-based and institutional settings as well as youths living in street settings, who constitute a particularly marginalized and overlooked population.^28^ The study was conducted in 1 county of western Kenya over almost 10 years. Our primary hypothesis was that living in institutional settings would be associated with higher HIV incidence and death compared with living in family-based settings but that living in either of those settings would be associated with lower HIV incidence and death compared with living in street settings.

## Methods

The Moi University College of Health Sciences and the Moi Teaching and Referral Hospital Institutional Research and Ethics Committee, the Indiana University Institutional Review Board, and the University of Toronto Research Ethics Board approved this study. Written informed consent for participation was provided by the head of household, the director of the charitable children’s institution, or, in the case of street-connected youths, by the district (now county) children’s officer. Individual written informed assent was provided by each child 7 years or older. Fingerprints were used for children and guardians who were unable to sign or write their names. This study followed the Strengthening the Reporting of Observational Studies in Epidemiology (STROBE) reporting guideline for cohort studies.

### Design and Setting

The Orphaned and Separated Children’s Assessments Related to Their Health and Well-Being (OSCAR) project was a 2-phase longitudinal cohort study. In-depth details about the OSCAR cohort have been previously reported.^29^ Phase 1 was conducted from 2010 to 2015 and phase 2 from 2016 to 2019.

The study was conducted in Uasin Gishu County, one of Kenya’s 47 counties, in the western highlands. Its capital, Eldoret, is home to the Moi University College of Health Sciences, the Moi Teaching and Referral Hospital, and the Academic Model Providing Access to Healthcare (AMPATH) program headquarters.^30^

### Participants

The study enrolled participants 18 years and younger between May 31, 2010, and April 24, 2013, with follow-up until November 30, 2019. The OSCAR cohort comprised participants from communities within 8 administrative locations in Uasin Gishu County, which included 300 randomly selected households (family-based settings) caring for children who were orphaned from all causes, 19 of 21 charitable children’s institutions (institutional settings) that were operating in the county at the time of study initiation, and a convenience sample of 100 children who were practicing self-care on the streets (street settings).^29^ Orphaned youths were defined as those with a biological mother, father, or both who had died. Separated youths were defined as those with a biological mother or father who was potentially alive but functionally not part of the child’s life as reported by the head of household. Street-connected youths were defined as those who spent most of their time (>75%) on the street during the night and/or day for at least the past 3 months.

### Independent Variables

The primary exposure of interest was care environment (institutional, family-based, or street), which was determined by a participant’s living circumstances at enrollment.^21^ Sociodemographic characteristics were ascertained through a clinical encounter and included age, sex, orphaned or separated status (maternal, paternal, or both), HIV status (positive, negative, or unknown), and time living in current care environment at baseline (<6 months, 6 months to <2 years, 2-5 years, >5 years, or all of life).

#### Outcomes

Primary outcomes were incident HIV and death. Counseling and testing for HIV was offered to all participants 18 months and older using rapid fingerstick assays administered by nationally certified HIV counselors. Children younger than 18 months were referred to the local HIV clinic for DNA testing to ascertain HIV status. All-cause death was ascertained at regular intervals by community health workers who visited participating households and documented deaths using standardized death reporting tools. Deaths among children in institutional settings were ascertained through annual assessments, which documented the outcomes of participants who were no longer living at the institutions. Deaths among street-connected youths were ascertained by the project social worker (who maintained extensive networks within the street youth community) and by physical tracing of children.

#### Data Sources

Data collection was conducted in situ at the participating charitable children’s institutions or at the OSCAR project clinic for participants living in family-based and street settings. Participants completed a standardized clinical evaluation annually (or semiannually for street-connected youths), and children 10 years and older completed an additional psychosocial evaluation annually.^31^ The clinical encounter was an enhanced well-child care visit that included a complete survey of physical history and a review of symptoms. Household-level data were obtained through annual site assessments administered by the project manager (for charitable children’s institutions) or community health workers (for participating households).^32^ Site assessments were not conducted for street-connected youths.

#### Bias

Community health workers were study staff dedicated to following up participants from households in the community to ascertain deaths and other issues on a quarterly basis throughout the lives of the participants. We used our relationships and networks with the charitable children’s institutions to ascertain outcomes of children within their environments who were no longer living in the institutions because they were older than 18 years. We conducted dedicated team-based outreach to other cities in Kenya to which street-connected children were known to have migrated, which enabled us to ascertain outcomes directly from participants.

We calculated effect estimates that were adjusted for the potential confounders of age and sex. We used competing risk regression analysis for HIV incidence because death is a competing risk for this outcome,^33^ and we used Cox survival models to assess death and time to incident HIV or death. The covariate for age was categorized as younger than 12 years vs 12 years and older to account for the onset of puberty and the increased probability of sexual activity after puberty. Robust SEs were calculated to account for clustering by care environment. In a sensitivity analysis, we assessed the effect of censor year to examine bias that may have occurred from differential follow-up times.

#### Study Size

Before study initiation, we conducted power and sample size calculations to estimate our power to detect 5%, 10%, and 15% differences in the probability of death. Our calculations assumed (1) a sample of 1110 children living in institutional settings and 305 children living in family-based settings, (2) a 3:1 ratio of children in institutional settings to children in family-based settings, (3) an approximately equal baseline risk for the outcome of interest among all children in the study, (4) a mean cluster size of 1.5 for families and 60 for institutions, (5) an intraclass correlation governing cluster effects of 0.20, (6) a loss to follow-up rate of 10%, and (7) a type 1 error rate of 2-sided α = .05. The calculations indicated that we had 86% to 99% power to detect the prespecified differences. Because the actual enrollment numbers exceeded expectations, the power was expected to be higher using the same assumptions.

### Statistical Analysis

For all study participants, demographic characteristics at enrollment were both summarized and stratified by care environment. For participants in family-based and institutional settings, care environment characteristics at the site assessment closest to enrollment were summarized at the participant level. We reported mean values with SDs for continuous variables and frequencies with percentages for categorical variables, both overall and by care environment. Youths who were not orphaned or separated were excluded from all analyses.

We conducted a survival analysis to assess the association of care environment with HIV incidence, death associated with any cause, and time to incident HIV or death. For each outcome, we assessed overall survival by care environment using Kaplan-Meier estimates of cumulative incidence and the *P* value from a log-rank test. Results from models were reported as hazard ratios (HRs) with 2-sided 95% CIs, and *P* < .05 was considered statistically significant. Participants with HIV-positive status at study enrollment were omitted from the analyses of HIV incidence and time to incident HIV or death. As a secondary analysis, we examined the association of sex and added an interaction term between care environment and sex to the same analyses that were conducted for direct comparisons by care environment. All analyses were performed using R software, version 4.0.1 (R Foundation for Statistical Computing).^34^

## Results

### Participant Sociodemographic Characteristics

The analysis included 2551 participants. Of those, 1230 participants were living in family-based settings, 1230 were living in institutional settings, and 91 were living in street settings (Table 1). The mean (SD) age at baseline was 10.4 (4.8) years, and 1321 participants (51.8%) were male; among street-connected youths, 70 participants (76.9%) were male. Higher proportions of participants living in charitable children’s institutions and street settings were double orphaned (1047 youths [85.1%] and 71 youths [78.0%], respectively), whereas only 487 youths (39.6%) living in family-based settings were double orphaned. At baseline, 1364 total participants (53.5%; 331 youths [26.9%] from institutional settings, 1017 [82.7%] from family-based settings, and 16 [17.6%] from street settings) had been living in their current care environment for 5 years or longer or all of their lives. Most street-connected participants (54 youths [59.3%]) had been living on the street for 6 months to 5 years. Additional characteristics of the 2474 participants with HIV-negative status at enrollment who were included in the analysis of HIV outcomes are available in eTable 1 in the Supplement.

**Table 1.** Baseline Participant Characteristics

Characteristic	No. (%)			
	Total	Institutional setting	Family-based setting	Street setting
Total participants, No.	2551	1230	1230	91
Age, mean (SD), y	10.4 (4.8)	10.3 (4.8)	10.3 (4.8)	14.5 (3.3)
Age group, y				
≤5	426 (16.7)	229 (18.6)	196 (15.9)	1 (1.1)
>5 to ≤10	684 (26.8)	300 (24.4)	377 (30.7)	7 (7.7)
>10 to ≤13	559 (21.9)	282 (22.9)	258 (21.0)	19 (20.9)
>13 to ≤16	563 (22.1)	285 (23.2)	250 (20.3)	28 (30.8)
>16	319 (12.5)	134 (10.9)	149 (12.1)	36 (39.6)
Sex				
Female	1230 (48.2)	568 (46.2)	641 (52.1)	21 (23.1)
Male	1321 (51.8)	662 (53.8)	589 (47.9)	70 (76.9)
Orphan status				
Double (maternal and paternal)	1605 (62.9)	1047 (85.1)	487 (39.6)	71 (78.0)
Maternal	214 (8.4)	82 (6.7)	128 (10.4)	4 (4.4)
Paternal	732 (28.7)	101 (8.2)	615 (50.0)	16 (17.6)
Time living in current care environment				
<6 mo	186 (7.3)	165 (13.4)	14 (1.1)	7 (7.7)
6 mo to <2 y	389 (15.2)	327 (26.6)	39 (3.2)	23 (25.3)
2-5 y	564 (22.1)	380 (30.9)	153 (12.4)	31 (34.1)
>5 y	414 (16.2)	281 (22.8)	122 (9.9)	11 (12.1)
All of life	950 (37.2)	50 (4.1)	895 (72.8)	5 (5.5)
Missing	48 (1.9)	27 (2.2)	7 (0.6)	14 (15.4)

The median follow-up time was 6.2 years (interquartile range [IQR], 2.0-7.4 years) among all participants, 3.1 years (IQR, 1.0-7.0 years) among participants in institutional settings, 6.9 years (IQR, 2.3-7.4 years) among participants in family-based settings, and 6.5 years (IQR, 2.0-8.1 years) among participants in street settings. Details regarding participants unavailable for follow-up are summarized in eTable 2 in the Supplement.

At baseline, 77 participants (3.0%) had HIV-positive status, including 53 youths (4.3%) in institutional settings, 23 youths (1.9%) in family-based settings, and 1 youth (1.1%) in a street setting (Table 2). A total of 31 youths (1.2%) acquired HIV infection during the study, 28 youths (1.1%) died, and 51 youths (2.0%) acquired HIV infection or died during the study (Figure). The HIV incidence was 2.06 cases per 1000 person-years (95% CI, 1.1-3.0 cases per 1000 person-years) for the total cohort, 2.2 cases per 1000 person years (95% CI, 0.5-3.8 cases per 1000 person-years) for youths in institutional settings, 1.2 cases per 1000 person-years (95% CI, 0.2-2.1 cases per 1000 person-years) for youths in family-based settings, and 15.5 cases per 1000 person-years (95% CI, 3.1-27.1 cases per 1000 person-years) for youths in street settings. The mortality rate was 1.7 deaths per 1000 person-years (95% CI, 0.8-2.6 deaths per 1000 person-years) for the total cohort, 0.3 deaths per 1000 person-years (95% CI, 0-0.9 deaths per 1000 person-years) for youths in institutional settings, 2.2 deaths per 1000 person-years (95% CI, 0.9-3.5 deaths per 1000 person-years) for youths in family-based settings, and 8.6 deaths per 1000 person-years (95% CI, 0-17.9 deaths per 1000 person-years) for youths in street settings. The time to incident HIV or death was 3.0 cases or deaths per 1000 person-years (95% CI, 1.1-4.9 cases or deaths per 1000 person-years) for youths in institutional settings compared with 3.4 cases or deaths per 1000 person-years (95% CI, 1.8-5.0 cases or deaths per 1000 person-years) for youths in family-based settings and 25.7 cases or deaths per 1000 person-years (95% CI, 10.1-40.1 cases or deaths per 1000 person-years) for youths in street settings.

**Table 2.** Factors Examined at Baseline and Last Follow-up

Factor	No./total No. (%)							
	Baseline				Last follow-up^a^			
	Total (N = 2551)	Institutional setting (n = 1230)	Family-based setting (n = 1230)	Street setting (n = 91)	Total (N = 2551)	Institutional setting (n = 1230)	Family-based setting (n = 1230)	Street setting (n = 91)
HIV status at baseline^a^								
Negative	2453/2551 (96.2)	1160/1230 (94.3)	1203/1230 (97.8)	90/91 (98.9)	2159/2189 (98.6)	946/957 (98.9)	1147/1158 (99.1)	66/74 (89.2)
Positive	77/2551 (3.0)	53/1230 (4.3)	23/1230 (1.9)	1/91 (1.1)	28/2189 (1.3)	9/957 (0.9)	11/1158 (0.9)	8/74 (10.8)
Missing	21/2551 (0.8)	17/1230 (1.4)	4/1230 (0.3)	0	2/2189 (0.1)	2/957 (0.2)	0	1/74 (1.4)
Died during study^b^								
Yes	NA	NA	NA	NA	28/2551 (1.1)	3/1230 (0.2)	16/1230 (1.3)	9/91 (9.9)
No	NA	NA	NA	NA	2523/2551 (98.9)	1227/1230 (99.8)	1214/1230 (98.7)	82/91 (90.1)
Death among those with HIV-negative status at baseline								
Yes	NA	NA	NA	NA	25/2453 (1.0)	3/1160 (0.3)	13/1203 (1.1)	9/90 (10.0)
No	NA	NA	NA	NA	2428/2453 (99.0)	1157/1160 (99.7)	1190/1203 (98.9)	81/90 (90.0)
Death among those with HIV-positive status at baseline								
Yes	NA	NA	NA	NA	3/77 (3.9)	0	3/23 (13.0)	0
No	NA	NA	NA	NA	74/77 (96.1)	53/53 (100.0)	20/23 (87.0)	1/1 (100.0)
Acquired HIV infection or died								
Yes	NA	NA	NA	NA	51/2551 (2.0)	12/1230 (1.0)	24/1230 (2.0)	15/91 (16.5)
No	NA	NA	NA	NA	2402/2551 (94.2)	1148/1230 (93.3)	1179/1230 (95.9)	75/91 (82.4)
Had >1 study visit								
Yes	NA	NA	NA	NA	2189/2551 (85.8)	957/1230 (77.8)	1158/1230 (94.1)	74/91 (81.3)
No	NA	NA	NA	NA	362/2551 (14.2)	273/1230 (22.2)	72/1230 (5.9)	17/91 (18.7)

Abbreviation: NA, not applicable.

^a^ Among those with more than 1 study visit.

^b^ A total of 3 street-connected youths died but did not have more than 1 study visit.

**Figure.**
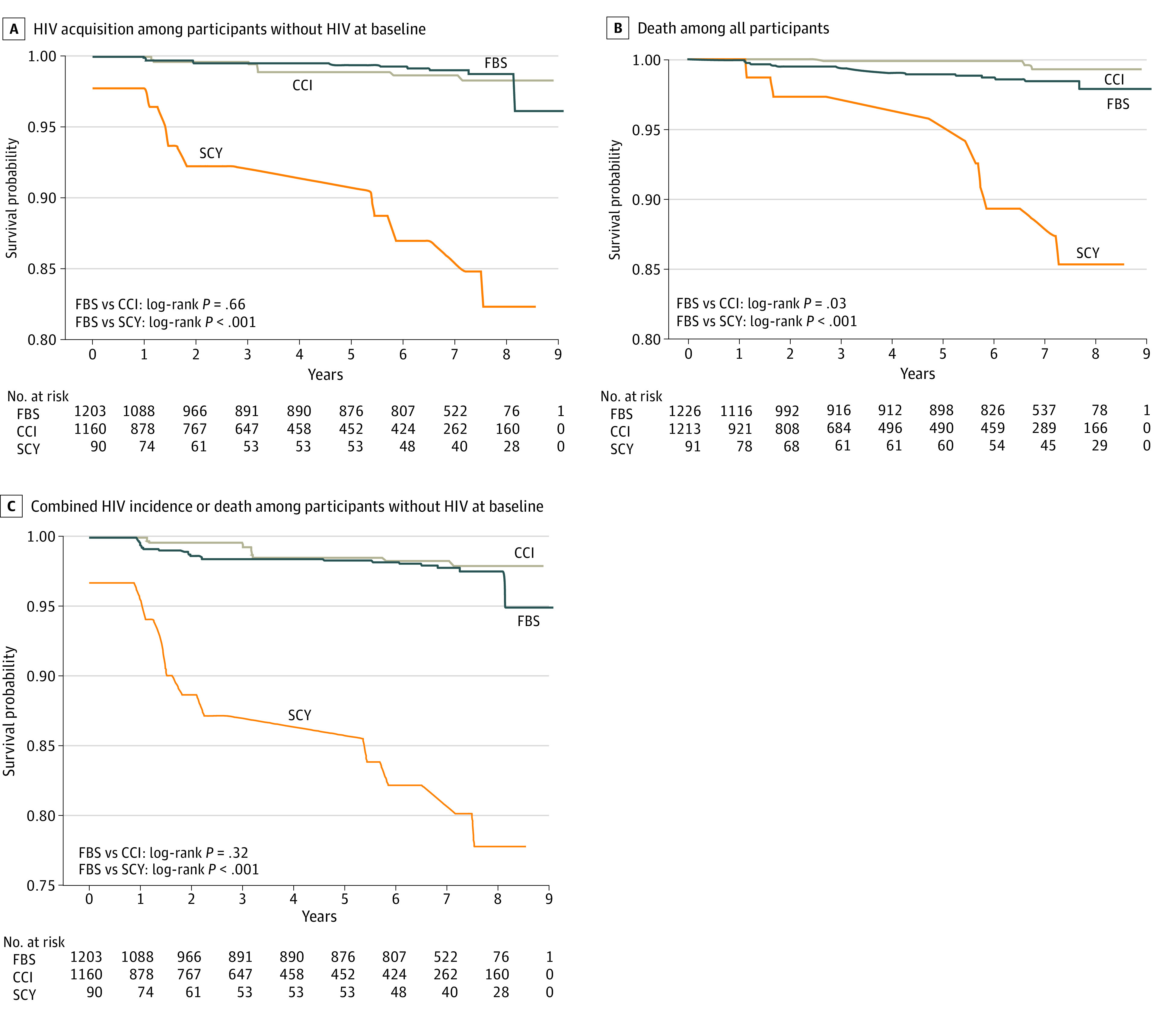
HIV Incidence, Death, and Time to Incident HIV or Death CCI indicates children living in a charitable children’s institution; FBS, children living in a family-based setting; and SCY, street-connected youths.

In the unadjusted analysis, living in an institutional setting vs a family-based setting was associated with a lower risk of death (HR, 0.29; 95% CI, 0.09-0.99; *P* = .047; log-rank *P* = .03) and no higher risk of HIV (HR, 1.33; 95% CI, 0.41-4.35; *P* = .64; log-rank *P* = .66); this association remained when the analysis was restricted to participants with HIV-positive status at baseline (log-rank *P* = .02) but not when restricted to participants with HIV-negative status at baseline (log-rank *P* = .10). After adjusting for sex, age, and baseline HIV status (Table 3), living in an institutional setting was not significantly associated with death compared with living in a family-based setting (adjusted HR [AHR], 0.26; 95% CI, 0.07-1.02; *P* = .05) or HIV incidence (AHR, 1.49; 95% CI, 0.46-4.83; *P* = .50). There were too few events to examine survival by sex. Compared with living in a family-based setting, living in a street setting was associated with death (AHR, 5.46; 95% CI, 2.30-12.94; *P* < .001), HIV incidence (AHR, 17.31; 95% CI, 5.85-51.25; *P* < .001), and time to incident HIV or death (AHR, 7.82; 95% CI, 3.48-17.55; *P* < .001). A significant difference in risk between male and female street-connected youths was found for HIV incidence (AHR, 0.28; 95% CI, 0.11-0.69; *P* = .006) but not death (AHR, 1.81; 95% CI, 0.81-4.01; *P* = .15). Among street-connected youths, there was no significant association with sex for the time to incident HIV or death (AHR, 0.69; 95% CI, 0.37-1.30; *P* = .25).

**Table 3.** Unadjusted and Adjusted HIV Incidence, Death, and Time to Incident HIV or Death^a^

Participant exposure	HIV incidence (n = 2551)^b^				Death (n = 2474)^c^				Time to incident HIV or death (n = 2474)			
	Unadjusted HR (95% CI)	*P* value	Adjusted HR (95% CI)	*P* value	Unadjusted HR (95% CI)	*P* value	Adjusted HR (95% CI)	*P* value	Unadjusted HR (95% CI)	*P* value	Adjusted HR (95% CI)	*P* value
Institutional vs family-based setting	1.33 (0.41-4.35)	.64	1.49 (0.46-4.83)	.50	0.29 (0.09-0.99)	.047	0.26 (0.07-1.02)	.05	0.70 (0.28-1.75)	.44	0.73 (0.29-1.86)	.51
Street vs family-based setting	13.33 (5.42-32.79)	<.001	17.31 (5.85-51.25)	<.001	7.76 (3.34-18.04)	<.001	5.46 (2.30-12.94)	<.001	8.84 (4.46-17.53)	<.001	7.82 (3.48-17.55)	<.001
Male vs female	NA	NA	0.28 (0.11-0.69)	.006	NA	NA	1.81 (0.81-4.01)	.15	NA	NA	0.69 (0.37-1.30)	.25
Baseline age ≥12 y vs <12 y	NA	NA	1.83 (0.74-4.51)	.19	NA	NA	2.02 (0.83-4.89)	.12	NA	NA	2.03 (0.99-4.16)	.05
Baseline HIV-positive vs HIV-negative status	NA	NA	NA	NA	NA	NA	6.84 (1.88-24.88)	.004	NA	NA	NA	NA

Abbreviations: HR, hazard ratio; NA, not applicable.

^a^ All models (adjusted and unadjusted) for HIV incidence and time to incident HIV or death omitted participants who had HIV-positive status at enrollment. The adjusted models were adjusted for age and sex.

^b^ Estimated using competing risks analysis, in which death was a competing risk for HIV infection.

^c^ The adjusted model for death was adjusted for baseline HIV status, age, and sex.

Because the median years of follow-up were different between participants living in institutional, family-based, and street settings, we performed a sensitivity analysis to explore the associations of different censor times with results. The IQR for follow-up times became more similar across groups as we censored the data at different points from 4 to 9 years after study initiation. After refitting the same models, the original results and conclusions were found to be relatively robust to censoring time (eTable 3 in the Supplement).

## Discussion

The findings of this cohort study suggest that among orphaned, separated, and street-connected children and adolescents in this region of Kenya, there was little or no association between care environment and HIV incidence or death, with the exception of street-connected youths, for whom the data suggested a substantial need for intervention. Our findings regarding the associations of institutional care compared with family-based care were inconsistent with those of a previous meta-analysis, which found that children in institutional care had worse outcomes than those in family-based care.^5^ There may be several explanations for this discrepancy. First, most of the studies included in the meta-analysis were conducted in Eastern Europe, and their results may therefore have been specific to geographic region. Second, the list of studies included in the meta-analysis was not published. Therefore, we could not be certain that data collected by the authors of the present study and other researchers working in low- and middle-income countries, which suggest that orphaned and separated children living in institutional environments have similar or even improved outcomes compared with children living in family-based settings, were included in the meta-analysis.^23,25,32,35,36,37,38,39,40,41^ Third, the comparator population used in the meta-analysis to conclude that institutionalization had adverse consequences comprised children who were being raised in a family or were included based on other standard norms derived from typically developing peers; thus, youths in the comparator population were not necessarily orphaned.^5^ Our data suggest that, when compared directly with orphaned and separated youths in family-based settings, the differences between care environments were minimal, and living in an institutional setting may be associated with some protective benefits.^23,24,31,32,42^ Our data are consistent with the findings of another study, which reported that the quality of care within care environments was more important to a child’s well-being than differences between care environment types.^25^

Our finding that living in a street setting vs a family-based setting was associated with HIV incidence and death was consistent with other, albeit limited, data from studies conducted in sub-Saharan Africa.^43,44,45^ These results highlight the substantial inadequacies of family-based care for many vulnerable young people, given that extreme poverty, family conflict, and child abuse and neglect are the primary reasons children migrate to the street.^28^ Although cash transfer programs for households caring for orphaned children have been implemented in Kenya and elsewhere, with generally positive impacts, these programs remain insufficient given the substantial need.^26,27,46,47,48,49,50,51,52,53^ The World Bank reported that only 18% of individuals in the lowest income quintile residing in low-income countries are covered by social safety nets.^54^ Given the already large number of children and adolescents living in the streets (who migrated there mostly because of poverty, family conflict, and violence or abuse^28^) and the lack of support for households caring for orphaned and separated children,^32,55^ caution from governments and other stakeholders is warranted when considering rapid and widespread deinstitutionalization.

As a last option, institutional environments, when regulated and monitored, have the potential to provide evidence-based care for larger numbers of children and adolescents, although their cost-effectiveness needs to be further investigated.^5,56^ A previous study described the ways in which the orphan dilemma, particularly in sub-Saharan Africa, requires a diversity of care environments to meet the needs of children and uphold their rights.^32^

### Strengths and Limitations

This study has strengths. First, it directly compares the HIV and survival outcomes of orphaned and separated youths in institutional environments with those in a random sample of households providing family-based care in the same geographic region, paying careful attention to sex equity. Second, it includes street-connected children and adolescents, who constitute a small but important population of especially high-risk and overlooked youths. Our data highlight that this population is in greater need of safety, care, and support relative to either of the other 2 groups. Third, this cohort study was powered to investigate the associations between care environment and death and followed up participants for almost 10 years. Fourth, HIV and death outcomes were ascertained by project staff and were not dependent on self-reporting.

This study also has limitations. First, the study is observational and cannot ascertain causality. Second, the study does not examine long-term outcomes; the median follow-up of participants living in institutional settings was shorter than that of participants living in family-based settings, likely because Kenya mandates the exiting of institutions at age 18 years (with rare exceptions for youths finishing secondary school). Third, the relatively low number of deaths and incident HIV cases prohibit us from reaching firm conclusions or adjusting for multiple covariates. Nevertheless, these data are among the only prospective data available about these important outcomes among highly vulnerable populations, and they provide contextually relevant information that suggests further study is warranted.

## Conclusions

Orphaned and separated children and adolescents living in institutional environments may have survival and HIV outcomes that are comparable with those living in family-based environments. In contrast, youths living in street settings had higher HIV incidence and death, with female sex being significantly associated with incident HIV. In a context in which the foster care system is inadequately developed and insufficiently monitored, creative and open-minded strategies are necessary to help care for the millions of children and adolescents in need. Policies and programs will need to maintain the best interests of children as their guiding principle, recognize and implement programs in which families can be better supported to care for orphaned and separated youths, and provide an adequate safety net when biological or foster families are not an option.

## Notes

Supplement. **eTable 1.** Comparison of Participants in the Cohort Study I Enrollment by Care Environment for All Participants and for Participants Who Were HIV-Negative at Enrollment **eTable 2.** Outcomes of Participants in the Cohort Study, Phase 1 and Phase 2 **eTable 3.** Results From Sensitivity Analysis Modifying the Year of Censor and Its Impact on the Proportional Hazards and Tests of Statistical Significance
