# Supplementary material for: Association of Care Environment With HIV Incidence and Death Among Orphaned, Separated, and Street-Connected Children and Adolescents in Western Kenya
Source: JAMA Netw Open. 2021 Sep 16;4(9):e2125365. doi: 10.1001/jamanetworkopen.2021.25365 (PMC8446813; doi:10.1001/jamanetworkopen.2021.25365)
Supplement: Supplement. — eTable 1. Comparison of Participants in the Cohort Study I Enrollment by Care Environment for All Participants and for Participants Who Were HIV-Negative at Enrollment eTable 2. Outcomes of Participants in the Cohort Study, Phase 1 and Phase 2 eTable 3. Results From Sensitivity Analysis Modifying the Year of Censor and Its Impact on the Proportional Hazards and Tests of Statistical Significance [file jamanetwopen-e2125365-s001.pdf]

Supplementary Online Content

Braitstein P, DeLong A, Ayuku D, et al. Association of care environment with HIV incidence and death among orphaned, separated, and street-connected children and adolescents in western Kenya. *JAMA Netw Open*. 2021;4(9):e2125365. doi:10.1001/jamanetworkopen.2021.25365

eTable 1. Comparison of Participants in the Cohort Study I Enrollment by Care Environment for All Participants and for Participants Who Were HIV-Negative at Enrollment

eTable 2. Outcomes of Participants in the Cohort Study, Phase 1 and Phase 2

eTable 3. Results From Sensitivity Analysis Modifying the Year of Censor and Its Impact on the Proportional Hazards and Tests of Statistical Significance

This supplementary material has been provided by the authors to give readers additional information about their work.

© 2021 Braitstein P et al. *JAMA Network Open*.

eTable 1. Comparison of Participants in the Cohort StudyI Enrollment by Care Environment for All Participants and for Participants Who Were HIV-Negative at OSCAR I Enrollment

The analysis of death incudes all participants, which the analysis for HIV incidence and HIV-free survival includes those HIV-negative at enrollment.

		Cohort for all-cause mortality outcome			Cohort for HIV acquisition and HIV or death outcomes		
**Variable**	**Value**	**CCI**	**FBS**	**SCY**	**CCI**	**FBS**	**SCY**
		n=1230	n=1230	n=91	n=1177	n=1207	n=90
Age (Years)	Mean (SD)	10.3 (4.8)	10.3 (4.8)	14.4 (3.3)	10.4 (4.8)	10.3 (4.8)	14.4 (3.3)
Age Group							
	<=5	229 (18.6)	196 (15.9)	1 (1.1)	214 (18.2)	195 (16.2)	1 (1.1)
	>5 & <=10	300 (24.4)	377 (30.7)	7 (7.7)	282 (24.0)	369 (30.6)	7 (7.8)
	>10 & <=13	282 (22.9)	258 (21.0)	19 (20.9)	271 (23.0)	250 (20.7)	19 (21.1)
	>13 & <=16	285 (23.2)	250 (20.3)	28 (30.8)	278 (23.6)	247 (20.5)	28 (31.1)
	>16	134 (10.9)	149 (12.1)	36 (39.6)	132 (11.2)	146 (12.1)	35 (38.9)
Gender							
	Female	568 (46.2)	641 (52.1)	21 (23.1)	539 (45.8)	629 (52.1)	20 (22.2)
	Male	662 (53.8)	589 (47.9)	70 (76.9)	638 (54.2)	578 (47.9)	70 (77.8)
Orphan Status							
	Double Orphan	1047 (85.1)	487 (39.6)	71 (78)	1000 (85)	479 (39.7)	70 (77.8)
	Maternal Orphan	82 (6.7)	128 (10.4)	4 (4.4)	77 (6.5)	126 (10.4)	4 (4.4)
	Paternal Orphan	101 (8.2)	615 (50.0)	16 (17.6)	100 (8.5)	602 (49.9)	16 (17.8)
Time with caregiver							
	Less than 2 years	492 (40)	53 (4.3)	30 (33)	467 (39.7)	52 (4.3)	30 (33.3)
	> 5 years	281 (22.8)	122 (9.9)	11 (12.1)	274 (23.3)	120 (9.9)	10 (11.1)
	2-5 years	380 (30.9)	153 (12.4)	31 (34.1)	363 (30.8)	149 (12.3)	31 (34.4)
	all his/her life	50 (4.1)	895 (72.8)	5 (5.5)	49 (4.2)	879 (72.8)	5 (5.6)
	Missing	27 (2.2)	7 (0.6)	14 (15.4)	24 (2.0)	7 (0.6)	14 (15.6)

© 2021 Braitstein P et al. *JAMA Network Open*.

eTable 2. Outcomes of Participants in the Cohort Study, Phase 1 and Phase 2

	Active**			Transitioned Out*			Dead			Lost to follow-up		
	CCI	FBS	SCY	CCI	FBS	SCY	CCI	FBS	SCY	CCI	FBS	SCY
**##Enrollment**	**1230**	**1230**	**91**	-	-	-	-	-	-	-	-	-
**End of phase 1**	982	1198	88	137	13	0	1	7	3	110	12	0
**###Eligible for Enrollment in phase 2**	**887**	**1198**	**88**	-	-	-	1	-	-	-	-	-
**Visit 1 in phase 2**	508	1022	54	125	128	0	1	7	6	254	41	28
**End of phase 2**	371	810	52	92	133	1	0	2	0	45	77	1
**Total** (Cumulative transitions, deaths and LTFU)				354	274	1	3	16	9	409	130	29

* Transitioned out includes repatriated home, adopted, aged out, married, moved away, expelled, employed elsewhere, run-away.

** Active includes completed study visit or missed the visit because they were temporarily unavailable (e.g. at school, on a trip, etc).

This is every participant who got enrolled and was part of our analysis.

This number excludes those who died at the end of phase 1 or before enrollment into phase 2 and those who were either lost to follow-upU or had transitioned out and did not participate in phase 2 of the study.

© 2021 Braitstein P et al. *JAMA Network Open*.

eTable 3. Results From Sensitivity Analysis Modifying the Year of Censor and Its Impact on the Proportional Hazards and Tests of Statistical Significance

Comparison	Year of Censor	Death	HIV Acquisition*	Death or HIV Acquisition*
CCI** vs FBS***	4	0.12 (0.01, 1.06) p=0.06	0.59 (0.16, 2.21) p=0.43	0.77 (0.31, 1.92) p=0.57
CCI vs FBS	5	0.12 (0.01, 1.00) p=0.05	0.75 (0.2, 2.73) p=0.66	0.74 (0.30, 1.82) p=0.51
CCI vs FBS	6	0.12 (0.01, 0.95) p=0.04	1.26 (0.29, 5.51) p=0.76	0.81 (0.30, 2.18) p=0.68
CCI vs FBS	7	0.29 (0.07, 1.17) p=0.08	1.24 (0.29, 5.31) p=0.77	0.73 (0.28, 1.92) p=0.53
CCI vs FBS	8	0.26 (0.07, 1.04) p=0.06	1.94 (0.57, 6.61) p=0.29	0.77 (0.30, 1.96) p=0.59
CCI vs FBS	9	0.26 (0.07, 1.04) p=0.06	1.49 (0.46, 4.83) p=0.50	0.74 (0.29, 1.87) p=0.52
SCY**** vs FBS	4	2.13 (0.48, 9.46) p=0.32	16.65 (4.17, 66.53) p<0.01	8.19 (3.12, 21.53) p<0.01
SCY vs FBS	5	2.99 (0.79, 11.29) p=0.11	19.52 (4.97, 76.67) p<0.01	7.70 (2.98, 19.94) p<0.01
SCY vs FBS	6	5.99 (2.21, 16.25) p<0.01	24.5 (7.46, 80.45) p<0.01	1.81(4.12, 23.34) 1.82p<0.01
SCY vs FBS	7	4.96 (1.90, 12.98) p<0.01	27.07 (8.09, 90.62) p<0.01	8.61 (3.71, 19.97) p<0.01
SCY vs FBS	8	5.83 (2.43, 13.99) p<0.01	33.97 (10.85, 106.31) p<0.01	8.89 (3.99, 19.81) p<0.01
SCY vs FBS	9	5.83 (2.43, 13.99) p<0.01	17.3 (5.84, 51.23) p<0.01	8.40 (3.75, 18.81) p<0.01

*Excludes those HIV-positive at enrolment

**CCI: Charitable Children's Institutions

***FBS: Family-based settings

****SCY: Street-connected children and youth

© 2021 Braitstein P et al. *JAMA Network Open*.
